# Pitfalls in Root Trait Calculations: How Ignoring Diameter Heterogeneity Can Lead to Overestimation of Functional Traits

**DOI:** 10.3389/fpls.2017.00898

**Published:** 2017-05-29

**Authors:** Laura Rose

**Affiliations:** Department of Geobotany, Faculty of Biology, University of FreiburgFreiburg, Germany

**Keywords:** functional root traits, root diameter, root morphology, root tissue density, specific root length, WinRHIZO^TM^

## Abstract

Specific root length (SRL) and root tissue density (RTD) are ecologically functional traits which are calculated from root length or volume and root dry weight. Both can be converted into each other using the root diameter assuming roots are cylindrical. The calculation of volume from length or length from volume is, however, problematic because samples of roots do usually not have a constant diameter. Ignorance of the diameter heterogeneity leads to an overestimation of length and an underestimation of volume if standard formulas are used. Here I show for two datasets that SRL and RTD are overestimated on average 67% for the two analyzed datasets, but up to 150%, if calculated from each other. I further highlight that the volume values for the total sample as provided by the commonly used software WinRHIZO^TM^ should only be used for objects with constant diameter. I recommend to use volume values provided for each diameter class of a sample if WinRHIZO^TM^ is used. If manual methods, like the line-intersect method, are used, roots should be separated into diameter classes before length measurements if the volume is calculated from length. Trait to trait conversions for whole samples are not recommended.

## Introduction

Root traits, such as specific root length (length per dry mass, SRL) and root tissue density (dry mass per volume, RTD) are used to functionally describe roots equivalent to the functional leaf traits specific leaf area and leaf dry matter content (Elberse and Berendse, 1993; Ryser, 1996; Ryser and Eek, 2000; Freschet et al., 2015). The SRL is typically positively related to nitrogen uptake rates (Reich et al., 1998) and negatively related to root life span (Ryser, 2006), while the RTD shows a positive correlation with root life span (Ryser, 1996; Eissenstat et al., 2000). They can further be indicative of resource availability as they respond to variation in nutrient (Leuschner et al., 2013; Freschet et al., 2015) and water (de Vries et al., 2016) availability. Both traits are thereby linked to nutrient and carbon cycling.

Specific root length and RTD are based on measurements of length (L) or volume (V) and root dry weight. Root length can be estimated with the line-intersect method (Tennant, 1975), which estimates the length of a root sample based on the number of intersections with grid-lines on a grid with a known area. Root volume can be obtained by quantifying the volume of liquid a root displaces (Archimedes’ principle, e.g., Birouste et al., 2014).

Although calculations of root length and volume (and SRL and RTD) and conversion of one to the other by using the diameter (D) appear to be easy if we assume that roots are cylindrical, problems arise because round roots do not necessarily have a constant diameter.

Ostonen et al. (2007) state that the SRL can be calculated from the RTD and diameter based on the conversion of length into volume assuming a cylinder:

(1) $$V=L\times {\left(\frac{D}{2}\right)}^{2}\times \;\pi$$

This formula is, however, problematic as soon as we have a shape with heterogenous diameter (Ryser, 2006). Because of the quadratic term, the influence of the part of the root with above average D will be greater than the influence of the part with below average D. In consequence, the length is overestimated and the volume underestimated if the parameters are calculated from each other. The same problem applies to samples that represent cohorts of flat round objects, like xylem vessels or bacteria colonies. If the diameters of vessels or colonies vary within a sample, the surface area of the total sample (TS) would be underestimated if the diameters were averaged before using trigonometric formulas. It follows that RTD is overestimated if it is calculated from SRL (underestimation of V) and SRL is overestimated if we calculate it from RTD. The problem does not apply to the root surface area which increases and decreases symmetrically if a cylinder gets thicker or thinner. Hence, specific root area is not discussed in this context.

Technical advances during the last 25 years provoked the development of digital image analysis software, which allows to calculate root length and volume from two-dimensional root images. The most commonly used commercial software designed to analyze images of cleaned roots is the WinRHIZO^TM^ series. It uses two-dimensional images to compute length, diameter, surface area and volume of root samples. The first two variables of interest in the output are the TS length and projected area, followed by its surface area, average diameter, length per soil volume and root volume. While length and projected area are measured based on pixel counts; surface area, diameter and volume are estimated ‘based on the assumption that roots are round’ (Régent Instruments Inc, 2013, Appendix PP. 129–130). The diameter is calculated as projected area per length and the volume is calculated from the total length and average diameter following Equation 1. This means that the TS volume is underestimated if the diameter of the root sample in one image is variable (**Supplementary Figure S1**). The output (except for the ‘Basic’ version) also provides values for length, surface area, projected area and volume for different (user-defined) diameter classes of the analyzed sample. These values are based on the continuous punctual diameter of each root fragment and are therefore not affected by variable diameter.

The aim of this perspective is to raise awareness for the potential severe overestimation of SRL and RTD when calculated from average diameter. Potential errors resulting from diameter variations within root samples or within sample cohorts of flat round objects are demonstrated using a hypothetical example dataset. Errors resulting from trait to trait conversion or from the naïve use of the software WinRHIZO^TM^ are demonstrated for European beech and four herbaceous species.

## Materials and Methods

### Example Data

The example data was constructed to represent four different root samples with an average diameter of 2 mm and a total length of 80 mm but different diameter distributions (**Table 1**). They could further be interpreted as samples of 80 flat round objects (e.g., xylem vessels), again with an average diameter of 2 mm but different diameter distributions. E1 represents a sample with constant diameter, and diameter variability increases from E2 to E4. The volume was then calculated for each sample from L and D for each fragment of constant diameter and summed to the TS value (E2 – E4). Further the length was calculated from V and D for each fragment and for the total volume. Subsequently, the divergence from the correct value for V and L that results from averaging the diameter was calculated.

**Table 1:** Examples for roots or cohorts of flat round objects with the same average diameter (weighted by length/number) and the same length/sample size but different diameter distributions.

	Diameter	Length (root) or Number (flat)	Volume (root) or Surface area (flat)	Length (from volume)	Divergence (%)
E1	2	**80**	**251.33**	**80**	
E2	1	10	7.85	10	
	2	60	188.50	60	
	3	10	70.69	10	
Total	2	**80**	**267.04**	**85**	**6.25**
E3	1	20	15.71	20	
	2	40	125.66	40	
	3	20	141.37	20	
Total	2	**80**	**282.74**	**90**	**12.5**
E4	1	40	31.42	40	
	3	40	282.74	40	
Total	2	**80**	**314.16**	**100**	**25**

*The root volume or the surface area for flat objects are calculated per fraction of equal diameter and subsequently summed up. The length is then calculated for the different fractions again or from the correct volumes (Total). The divergence is the percentage of underestimation of volume (reference 251) or overestimation of length (reference 80) calculated following Equation 4. E1 represents constant diameter or results based on the average diameter. Bold values indicate total sample values.*

### Root Processing

I analyzed one dataset of beech (*Fagus sylvatica* L.) fine roots (*n* = 204) and one dataset of herbaceous (*Veronica beccabunga* L., *Potentilla argentea* L., *P. recta* L., *P. tabernaemontani* Aschers.) fine roots (*n* = 18). All plants were grown in containers and younger than 1 year.

The roots were cleaned by washing them over a sieve with a mesh size of 0.2 mm. Root samples were randomly chosen from roots thinner than 2 mm for beech while the whole root system of each of the herbaceous plants was analyzed. Each sample was scanned in water with a flatbed scanner (Epson Perfection V700 Photo, SEIKO EPSON CORP., Japan, resolution 400 dpi). Subsequently the images were analyzed using the software WinRHIZO^TM^ Reg 2013e (Régent Instruments Inc., Canada). The pixel classification threshold was set to 144 and the diameter classes were set as: 0 – 0.1, 0.1 – 0.2, 0.2 – 0.3, 0.3 – 0.4, 0.4 – 0.5, 0.5 – 0.6, 0.6 – 0.7, 0.7 – 0.8, 0.8 – 0.9, 0.9 – 1.0, 1.0 – 1.1, 1.1 – 1.2, 1.2 – 1.3, 1.3 – 1.4, 1.4 – 1.5, 1.5 – 1.6, 1.6 – 1.7, 1.7 – 1.8, 1.8 – 1.9, >1.9 mm. Each sample was dried (70°C, 72 h) and weighed to allow for calculations of SRL and RTD.

### Analyses

Firstly, I calculated both SRL and RTD from the summed length and volume values provided for the diameter classes (SRL_DC_, RTD_DC_) and the dry weight for each sample. Secondly, I calculated SRL and RTD both from the TS values of L and V and the dry weight (SRL_TS_, RTD_TS_). I further calculated the RTD from SRL_DC_ and vice versa (SRL_TRAIT_, RTD_TRAIT_) following Equations 2 and 3 (**Supplementary Figure S1**).

(2) $$SRL=\frac{1}{RTD\times D^{2}}\times \frac{\text{4}}{\;\pi }$$

(3) $$RTD=\frac{1}{SRL\times D^{2}}\times \frac{\text{4}}{\;\pi }$$

For the trait from trait calculations I used the mean diameter weighted by length calculated from the projected area and the length of each WinRHIZO^TM^ diameter class.

Subsequently, I quantified the divergence between the values of SRL_TS_, RTD_TS_, SRL_TRAIT_ and RTD_TRAIT_ and the corresponding values of SRL_DC_ or RTD_DC_ as percentage of the SRL_DC_ or RTD_DC_ values (Supplementary Data S1). E.g.,

(4) $$Divergence(RTD_{TRAIT})=\frac{RTD_{TRAIT}-RTD_{DC}}{RTD_{DC}}\times 10$$

One sample *t*-tests performed with R version 3.2.3 (R Core Team, 2015) were used to test whether the divergence of the different trait values was significantly greater than 0 for beech and herbaceous species separately (Bonferroni corrected for 8 tests). A Wilcoxon test was used to test for a significant difference between the divergences of the trait based values of beech and the herbaceous species. The difference between the divergence of RTD_TS_ and RTD_TRAIT_ from RTD_DC_ was analyzed with a paired *t*-test.

## Results

### Example Data

The example data showed an increase of root volume with increasing magnitude of variation in root diameters (E1 < E2 < E3 < E4, **Table 1**). Averaging the diameter before volume calculation led to an underestimation of the volume. Likewise, calculating the length based on the volume of the different roots assuming constant average diameter resulted in overestimation the length of roots in E 2, 3, and 4. The magnitude of over- or underestimation depended on the grade of diameter variability (E2 < E3 < E4) and ranged between 6.25 and 25% in this dataset.

### Root Traits

**FIGURE 1:**
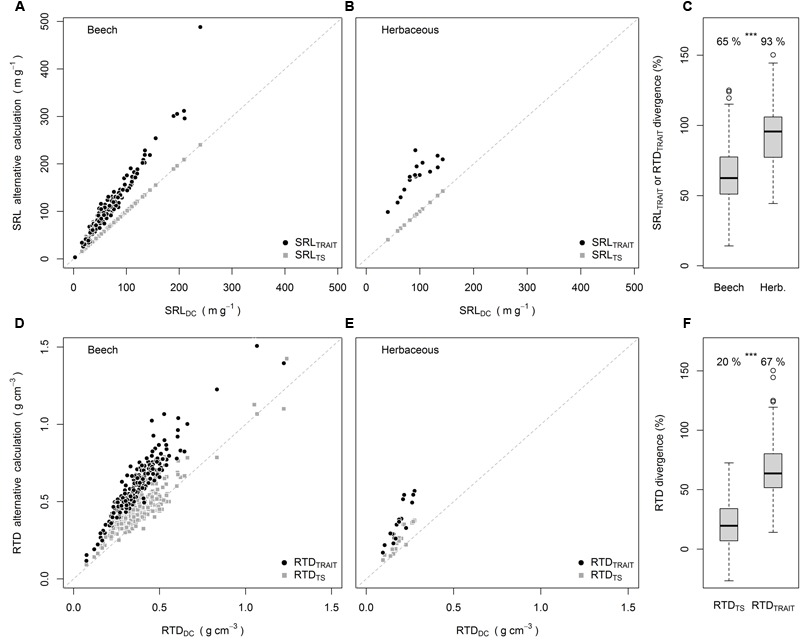
SRL_TRAIT_ and SRL_TS_ plotted against SRL_DC_ for beech **(A)** and herbaceous species **(B)**, divergence of SRL_TRAIT_ and RTD_TRAIT_ from SRL_DC_ and RTD_DC_ **(C)**, RTD_TRAIT_ and RTD_TS_ plotted against RTD_DC_ for beech **(D)** and herbaceous species **(E)**, average divergence of RTD_TS_ and RTD_TRAIT_ from RTD_DC_ **(F)**. Numbers in **(C,F)** are means, ^∗∗∗^ indicate significant differences [*P* < 0.001, Wilcoxon-test **(C)**, paired *t*-test **(F)**] -between groups. All means in **(C,F)** were significantly greater than 0 (*P* < 0.001, one sample *t*-test).

The divergence between SRL_TS_ and SRL_DC_ was significantly greater than 0 but only by 0.1% (analysis not shown). This should not be considered as ecologically meaningful, as also indicated by the SRL_TS_ values lying on the 1:1 line in **Figures 1A,B**. When the SRL was calculated from RTD it differed not only statistically from SRL_DC_, but also by an ecologically meaningful magnitude of +65% for beech and +93% for the herbaceous species (**Figures 1A–C**). Exactly the same divergence was found between RTD_TRAIT_ and RTD_DC_, which is a consequence of Equations 2 and 3. For RTD, however, there was also a significant divergence between RTD_TS_ and RTD_DC_ (20% higher values, **Figures 1D–F**). The divergence of RTD_TRAIT_ from RTD_DC_ (67%) was significantly higher than the divergence between RTD_TS_ and RTD_DC_ (**Figure 1F**). Further, for 15% of the samples RTD_TS_ was smaller than RTD_DC_.

## Discussion

The example data clearly demonstrated the potential error resulting from not taking the exact diameter of single cylindrical root segments into account. The same mathematical relationship is causing the divergences of SRL_TRAIT_ or RTD_TRAIT_ from SRL_DC_ or RTD_DC_. The root analyses show that the calculation of SRL or RTD from the diameter and the corresponding trait leads to significant overestimation of the respective trait. The magnitude of the introduced error depends on how much the diameter varies around the average, as also shown by the theoretical examples. For the root datasets, the error introduced was greater for the herbaceous species than for beech suggesting greater within sample diameter heterogeneity for the herbs. This shows that we have to be especially careful with our assumptions if we compare root traits of different groups or treatments that potentially differ in diameter distributions. Although Equation 2, as presented by Ostonen et al. (2007), is theoretically correct, it is dangerous if applied to roots. While the assumption that roots are round might very well be valid, the second assumption underlying the equation is that root diameters are constant within a sample. This assumption was not valid for the two datasets presented.

The small but significant difference between SRL_DC_ and SRL_TS_ resulted from differences in the WinRHIZO^TM^ length measurements for the whole sample and the single diameter classes within a sample but is independent of diameter measurements or averaging. For RTD, however, error sources are more complex. RTD_TRAIT_ is calculated from the weighted mean diameter while WinRHIZO^TM^ uses the whole sample average diameter to compute the TS volume used for RTD_TS_. The average diameter was 18% higher than the length-weighted mean diameter for beech and herbaceous samples combined (data not shown). Accordingly, the difference between RTD_DC_ and RTD_TRAIT_ results from averaging the diameter before volume calculations while the difference between RTD_DC_ and RTD_TS_ resulted from diameter averaging and the discrepancy between the two diameter measures (**Supplementary Figure S1**). A linear model revealed that 77 and 15% of the RTD_DC_ – RTD_TS_ divergence was explained by the RTD_DC_ – RTD_TRAIT_ divergence and the difference between the two diameter estimations respectively (analysis not shown). The higher average sample diameters explain the rare cases of underestimation of the RTD_TS_ compared to RTD_DC_. Which of the two diameter measures is more accurate is not subject of this perspective, but recommendations for testing diameter accuracy are provided by Bauhus and Messier (1999).

The most often analyzed root trait apart from average diameter is SRL. Luckily, root length is also the easiest value to measure. For though we introduce an error if we calculate SRL from RTD it is probably more often calculated from length. Measuring the root volume is, however, more complicated and calculating it from length is therefore tempting. WinRHIZO^TM^ 2013 (as well as older and more recent versions, Régent Instruments Inc, 2016) provides global values of volume for the TS based on the average diameter. Consistent with Equation 1, these values are also only correct for samples with constant diameter and therefore should not be used if that assumption is not valid. If WinRHIZO^TM^ is used for root volume estimates, the problem can be solved by adding up volumes of the different diameter classes. In this case it does not matter how many classes are chosen (analysis not shown) because the values are based on continuous punctual diameters and not on class averages (Régent Instruments Inc, 2013). This is, however, not possible for the ‘Basic’ version of the software, which only offers global values.

As already stressed by Ryser (2006), irrespective of the method used for measurements it is important to know the diameter and length of root segments for which volumes are calculated or vice versa. This could be achieved for manual measurements (e.g., line intersect method, Tennant, 1975) by first sorting roots into diameter classes and then estimating the length for each class. In this case the magnitude of underestimation of the volume would depend on the precision and number of the diameter classes. Alternative methods to accurately determine RTD such as the Archimedes method or estimates based on the root dry matter content are discussed by Birouste et al. (2014).

The assumption of constant diameter has to be considered in any area of biology that is analyzing cohorts of objects with heterogeneous diameters. We would, for example, underestimate the total area covered by termite mounds in a landscape or of bacteria colonies in a petri dish as well as the area of xylem vessels in wood, if we would calculate them based on the average diameter of a sample. Hence, beware of heterogeneity!

## Author Contributions

LR designed the study, collected and analyzed the data and wrote the manuscript.

## Conflict of Interest Statement

The author declares that the research was conducted in the absence of any commercial or financial relationships that could be construed as a potential conflict of interest.

## Supplementary Material

FIGURE S1 **Flow chart of different SRL and RTD calculations and potential error sources**.
