# Supplementary figures and images for: Pitfalls in Root Trait Calculations: How Ignoring Diameter Heterogeneity Can Lead to Overestimation of Functional Traits
Source: Front Plant Sci. 2017 May 29;8:898. doi: 10.3389/fpls.2017.00898 (PMC5447056; doi:10.3389/fpls.2017.00898)

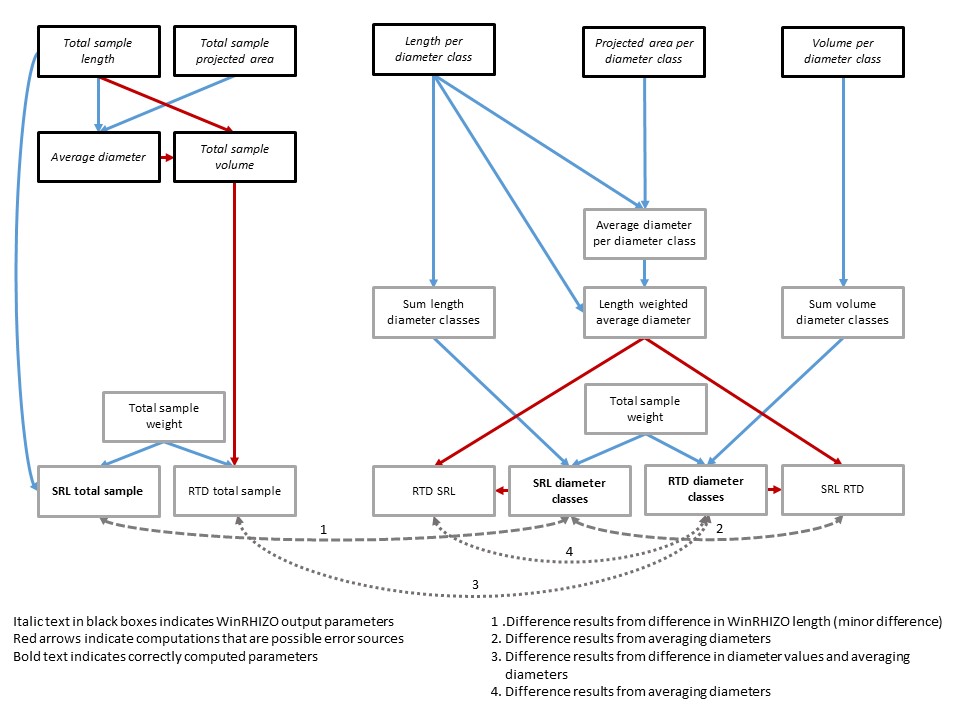

Supplement: FIGURE S1 — Flow chart of different SRL and RTD calculations and potential error sources. [file Image_1.JPEG]
